# A Dynamic Approach to Rebalancing Bike-Sharing Systems

**DOI:** 10.3390/s18020512

**Published:** 2018-02-08

**Authors:** Federico Chiariotti, Chiara Pielli, Andrea Zanella, Michele Zorzi

**Affiliations:** 1 Department of Information Engineering, University of Padova, 35131 Padova PD, Italy; chiariot@dei.unipd.it (F.C.); piellich@dei.unipd.it (C.P.); zorzi@dei.unipd.it (M.Z.); 2 Human Inspired Technologies (HIT) Research Center, University of Padova, 35131 Padova PD, Italy

**Keywords:** bike sharing, Smart Cities, dynamic rebalancing

## Abstract

Bike-sharing services are flourishing in Smart Cities worldwide. They provide a low-cost and environment-friendly transportation alternative and help reduce traffic congestion. However, these new services are still under development, and several challenges need to be solved. A major problem is the management of rebalancing trucks in order to ensure that bikes and stalls in the docking stations are always available when needed, despite the fluctuations in the service demand. In this work, we propose a dynamic rebalancing strategy that exploits historical data to predict the network conditions and promptly act in case of necessity. We use Birth-Death Processes to model the stations’ occupancy and decide when to redistribute bikes, and graph theory to select the rebalancing path and the stations involved. We validate the proposed framework on the data provided by New York City’s bike-sharing system. The numerical simulations show that a dynamic strategy able to adapt to the fluctuating nature of the network outperforms rebalancing schemes based on a static schedule.

## 1. Introduction

One of the most promising applications of the emerging Internet of Things (IoT) paradigm is Smart Cities [1]: the myriad sensors and actuators deployed in a modern city can provide many novel services to citizens and institutions and make the existing ones more efficient and user-friendly.

Bike sharing is a perfect example of a Smart City-enabled service. Although bike-sharing schemes have existed since the 1960s [2], issues with theft, vandalism and wrongful usage prevented their widespread adoption [3] until the arrival of smart biking systems. With the possibility of electronically unlocking bicycles and identifying users, smart bike-sharing systems solved or mitigated these issues: by requesting users’ credit card information before lending the bikes, bike-sharing companies can charge users for damages or theft, with a strong deterrent effect. The use of technology also allowed cities to provide a better service, embedding sensors to obtain real-time data, which can be used to plan and manage the bike-sharing docking stations and adapt to the needs of the users. Many services provide live maps of the available bikes, and ways to detect broken bikes remotely to quickly repair or substitute them are being studied [4]. Over the past few years, bike-sharing schemes were implemented in most major world cities, with almost universal success. Some of the biggest bike-sharing services in the world are in Barcelona, Paris, London, Hangzhou, Taiyuan and Shanghai in China, New York City, and Montreal. The usage data were easily recorded and used to improve the service and increase the number of users [5,6], reducing traffic and pollution. The abundance of data, sometimes including GPS tracking of the bikes’ trajectories, has led to new opportunities for Smart City management, such as, for example, the planning of new bike lanes to cover the most common routes [7].

In May 2013, New York City launched CitiBike, the largest bike-sharing system in North America. The CitiBike bike-sharing network consists of more than 700 docking stations in Manhattan, Brooklyn and Jersey City, with tens of thousands of rides every day. Despite such large numbers, the service is not always satisfactory. For example, the massive use of the service by commuters results into the quick depletion of the stations in residential areas in the morning hours, and the rapid exhaustion of the available stalls in the docking stations in commercial areas, so that users cannot deposit more bikes. In addition, stations at the top of slopes are more likely to be empty, as users are less keen on cycling uphill. This disparity highlights the importance of finding smart ways to manage large bike-sharing systems, and is reflected by the high rate of negative reviews of the service on TripAdvisor (36% of users rate CitiBike as “average” or lower) and by competitors’ recent experiments with “dockless” bikes that can be parked anywhere and unlocked with a smartphone app.

An effective way to improve bike-sharing systems consists of relocating bikes from overcrowded stations to those with a shortage of bikes. This technique is commonly known as *rebalancing*. Operators can, e.g., deploy a fleet of trucks to pick up and drop off bikes at different stations to balance the network. The trucks can all depart from the same location, or be distributed in different depots, depending on the size of the network and the operational costs of the service.

The design of an efficient rebalancing scheme, then, requires addressing two major challenges: predicting the demand for bikes and stalls in the docking stations in the different locations covered by the service, and selecting the most efficient way to relocate the bikes in order to best satisfy such a demand. Rather than performing bike rebalancing based on a static scheme, as is common in practical bikesharing services, in this study, we propose a *dynamic rebalancing* scheme that aims at increasing the users’ satisfaction by minimizing the probability of *service failures*, i.e., the chance that a user experiences an unsatisfactory service because of the unavailability of bikes or stalls in the docking stations. We model the occupancy of each station as a Birth-Death Process (BDP), with time varying birth and death rates (i.e., arrivals and departures, respectively). In this way, we are able to estimate how long a station will be self-sufficient before running out of bikes or available stalls, knowing its initial state. Periodically, we use the knowledge about such expected survival times to decide whether rebalancing is necessary. Clearly, employing a fleet of vehicles to rebalance the system has a monetary cost that counterbalances the improvement in the network conditions by moving bikes among stations. This cost also depends on the covered distance, as this affects the fuel consumption and the time required for the rebalancing operations. These are important factors that need to be tuned carefully when designing a rebalancing strategy because they play a role in deciding when to perform the bike relocation.

To gauge the performance of our scheme, we use three different metrics: the fraction of time the system is out of service (empty or full stations), the number of daily rebalancing operations, and the daily distance covered by the rebalancing trucks. Our scheme is compared with the benchmark case in which no relocation is done, and a static rebalancing scheme from the literature that rebalances bikes twice a day at preset times [8]. All the numerical evaluations are based on the dataset of the CitiBike system. In particular, we use the data collected over the past four years, during which the bike-sharing system grew in both number of stations and available bikes. The observed trips are a lower bound for true demand, as empty or full stations prevent users from taking or returning bikes, respectively. In addition, there may be unexpected events such as broken or stolen bikes that affect the bike availability, or special events in the city that make the bike demand differ from its usual pattern. Nonetheless, although the numerical results may be altered by the censoring problem, they are still representative of the positive impact the rebalancing has on the bike-sharing system. Furthermore, the framework we propose is general and can be readily adapted to new data. It is also an effective tool to identify the critical stations and locations so as to improve the bike-sharing systems by adding bikes at selected stations and increasing the station density in the critical areas. For a deeper analysis of the dataset, with considerations on other patterns such as weather and distance from mass transit stops, we refer the reader to our related work in [9], in which we compare the results from New York City with another dataset from a smaller system.

The rest of this work is organized as follows. Section 2 gives an outline of the state of the art on Smart Cities, bike sharing and rebalancing optimization. Section 3 presents our model of the bike-sharing problem, while Section 4 introduces the CitiBike dataset and the processing to derive the model parameters from it. Section 5 describes the performance of our dynamic rebalancing scheme. Finally, Section 6 contains our conclusions and some possible avenues of future research on the subject.

## 2. Related Work

Over the last few years, bike-sharing systems have gained a lot of momentum and become an active field of research, bolstered by the availability of real data from publicly open bike-sharing services in many cities. A comprehensive survey on the state of the art literature about bike-sharing systems can be found in [10], while [11] provides a history of bicycle sharing since 1965, when the city of Amsterdam launched the first bike-sharing program. In this section, we first introduce the possibilities of bike sharing as an integrated Smart City service. Then, we propose an overview of the two major branches of the scientific literature on bike-sharing system optimization, namely, the analysis of historical data to infer the demand, and rebalancing optimization techniques to limit system failures and user dissatisfaction.

### 2.1. Bike Sharing as a Smart Service

Bike sharing is a key component of future Smart Cities: bike-sharing services offer more flexibility than standard public transportation while also reducing both traffic and its impact on the environment [12] and improving public health [13]. According to an American Planning Association report [14], bike-sharing programs in North America significantly decreased subscribers’ car use, and bike sharing in Hangzhou, China, reportedly decreased CO${}_{2}$ emissions by 70 thousand tons a year. Like other mobility services, bike sharing provides a double benefit to Smart Cities: in addition to the direct improvements caused by the service, the data that the system gathers can be used to build models of citizens’ behavior and make more informed policy decisions, as well as providing a direction in which the Smart City can grow organically. Since Smart Cities are “systems of systems” [15], mobility is a key component and the integration of bike sharing in a Smart Mobility framework [16] can help achieve the goals of both the transport system and the city as a whole. This integration between data, services and communications has been proposed as the key idea of the SymbioCity paradigm [17].

An integrated approach to public transport, with car- and bike-sharing schemes supplementing standard bus and light rail systems, can improve transit times and encourage the shift towards a more sustainable urban mobility [18]. A study on two American cities [19] has found that bike-sharing is an alternative to public transport in high-density areas where distances are short, while the independence of these systems from fixed schedules and the higher density of stations can complement the deficiencies of traditional mass transit systems in lower-density areas, creating the missing “last mile” connection. The possibility of using e-bikes to increase the viable range of the system is also being explored [20].

Bike sharing also has an impact on urban planning: the presence of bike lanes [21,22] and urban zoning [23] has a strong effect on ridership, and future urban planning in Smart Cities should take this into account, both using bike sharing as a sustainable and flexible way to connect citizens and encouraging the transition from a car-oriented perspective to a more integrated approach to transportation, zoning and urban design [24]. Although there are several trade-offs and political issues that need to be tackled to shift towards a smart and sustainable mobility [25], research is focusing on formulating principles to guide urban design and help in the transition [26]: a key component of future mobility is the physical separation of fast, high mass vehicles such as cars and trucks from bikes, pedestrians, and other slow, low mass vehicles, increasing both safety and efficiency.

### 2.2. Traffic Analysis and Rebalancing

Recently, researchers have put a lot of effort in analyzing bike-sharing usage data to build models and frameworks that help improve the system. In [27], the bike demand at each station is modeled as a queuing system with finite capacity, and the service level requirements are then found using the transient distribution of the bikes’ availability and exploiting Kolmogorov forward equations [28]. This approach is similar to that proposed in this work. However, rather than determining a lower and upper bound for the target inventory level, we compute the occupancy state that maximizes the survival time of a station (i.e., the time before the stalls are either all empty or all full). Furthermore, our model is more flexible, being able to accommodate non stationary demand patterns.

Another efficient tool to extrapolate valuable information from real datasets is represented by machine learning. For example, Ref. [29] uses a neural network to learn the bike traffic pattern directly from the observed data, and also includes information such as the weather conditions, which are often troublesome to consider in an analytical model. The main drawback of this approach is that it requires a customized neural network for each bike-sharing system, whereas parameterized analytical models can be readily adapted to different bike-sharing systems.

Traffic analysis is also useful to construct clusters of stations with similar demand patterns [30], which may help the rebalancing optimization.

Although the historical data analysis gives insights on the bike demand, the heterogeneous user behavior and unexpected events make it challenging to accurately predict the traffic pattern [31]. Thus, external intervention is required to keep the system operational and limit failures due to the unavailability of bikes or docks, as this would have a negative impact on the users. A possibility consists in developing pricing strategies to incentivize users to return bikes to the least loaded among the closest stations [32,33]. However, pricing mechanisms may not be sufficient to achieve self-rebalancing of the system, while being annoying for the users. Hence, the majority of the existing bike-sharing systems employ a fleet of vehicles that pick up and move bikes between stations.

The most common rebalancing approaches can be classified as *static*, where the rebalancing is performed according to a predetermined schedule, likely when the system use is minimum (e.g., at night); or *dynamic*, in which the rebalancing occurs during the daytime, when needed.

In the literature, static rebalancing problems are often tackled through Mixed Integer Programming (MIP) techniques. For example, Ref. [34] uses a branch-and-cut algorithm on a relaxed MIP model together with a taboo search to obtain upper bounds to the NP-hard problem of redistributing bikes among stations with the minimum traveling distance. A similar method is used by Ho and Szeto in [35] inside an iterative procedure, resulting in a better solution. Several MIP techniques are used in [36], where a convex penalty objective function aims at minimizing both the user dissatisfaction and the costs of moving the vehicle fleet. The numerical experiment shows significant service quality improvements for networks with up to 100 stations with two repositioning vehicles. The authors also take into account the time needed to load and unload bikes. Constraint programming is used in [37], where a large neighborhood search approach is used to tackle the problem of balancing the bike-sharing system. Ref. [38] proposes a three-step mathematical programming based heuristic, which consists of clustering stations based on geographical and inventory considerations, and then constructing an appropriate traversal route. Differently from other approaches, rebalancing is not limited within clusters, but the routing vehicles can travel through a sequence of clusters. Similarly, Ref. [27] proposes a static rebalancing scheme where each vehicle is assigned a ’self-sufficient’ cluster of stations. Stations are in fact grouped in such a way that the target network configuration can be achieved by performing only within-cluster pickups and deliveries. Clusterization is useful to organize rebalancing paths more easily, but adopting such a technique in a dynamic rebalancing context is not trivial, since each rebalancing operation may involve different stations and clusters should continuously be identified. In [39], Dell’Amico et al. propose a metaheuristic that uses a destroy and repair approach to solve the static rebalancing problem, improving on the simple branch-and-cut approach that the same authors presented in [40], both in the quality of the solution and in the convergence time. These works are particularly interesting, as they consider the variability of the demand for bikes during the day and the different features of the stations, distinguishing between three different types of stations with similar traffic patterns.

Static approaches, however, may not be sufficient to avoid network failures during the day. To overcome this issue, dynamic rebalancing aims at redistributing bikes throughout the day according to the current network state. Clearly, this is much more challenging, since it includes a scheduling component based on the users’ activity during the rebalancing operation, and also a routing problem.

In [41], upper and lower bounds for the solution of a pickup-and-delivery problem are obtained by using Dantzig–Wolfe [42] and Benders [43] decompositions, respectively, but the study does not deal with a time-varying demand and, even in this simple scenario, the optimality gap of the approximation is large. An exact solution to the static rebalancing problem using Benders’ cuts for given target levels is instead provided in [44].

In [45], the loading instructions for the rebalancing vehicles are derived from an optimization function that weighs the unfulfilled user demand with the target filling level. Furthermore, each truck is assigned a capacity so that there is a maximum number of bikes it can load. An accurate simulation model is proposed in [29]: it simulates the bike-sharing system in space and time to determine the optimal repositioning flows and time intervals between relocation operations, by explicitly considering the route choice for trucks among the stations. Some studies also determine the optimal size of the rebalancing fleet [46]. A Markov Decision Process (MDP) is used in [47] to solve a stochastic inventory routing problem for bike-sharing systems. The objective is to minimize the number of expected violations of *due dates*, where a due date is the deadline within which a station has to be served by a rebalancing vehicle in order to satisfy the requests and, hence, avoid failures. The paper shows the importance of using both long-term and short-term relocation strategies. The former tries to estimate the target level that can deal with predicted demand, while the latter assigns a priority level to each station based on its urgency of being rebalanced. The short-term approach is similar to the one proposed in this paper, since we dynamically serve only those stations that are going to experience a failure in a short time horizon. An interesting framework is presented in [8], where rebalancing comprises two different strategies to tackle the bike imbalance during night and day, respectively: (i) static rebalancing overnight to move the network to an optimal configuration that minimizes the probability of stations becoming either empty or full in the following day, and (ii) clustering optimization to handle rush-hour usage and ensure that users are never too far from an available bike or dock. The joint use of the two strategies brings a larger improvement than considering the nightly rebalancing only. This result encouraged us in choosing a dynamic approach.

For a more detailed discussion on the rebalancing problem and other open research issues in bike-sharing systems, we refer the reader to [10,48].

To the best of our knowledge, our work is the first to consider a dynamic rebalancing scheme that can minimize the time during which stations are unable to meet user demand while considering the full complexity of the time-varying demand. Most previous efforts in the literature concentrate on finding the optimal route to rebalance bikes at fixed times, while we consider both whether the system should be rebalanced and which stations need it most, avoiding unnecessary trips.

## 3. System Model

We define the network of bike-sharing stations as a fully connected directed graph $G=(V,E^{2})$, where V≜{1,⋯,V} is the set of stations, and $E^{2}=\left(E,E\right)$ with E⊆V×V is the set of edges between stations. Note that each link is counted twice to allow for different costs between two stations according to the direction of the path. Each node v∈V is characterized by its capacity $M_{v}\in N$, i.e., the total number of bike stalls at the station, and its current occupancy $m_{v}\left(t\right)\in M_{v}≜\left\{0,1,\cdots ,M_{v}\right\}$, i.e., the number of bikes available at time *t*. The edges are weighed by a distance metric between stations, denoted as $d(v_{i},v_{j})$. As the graph is fully connected, each station can be reached from any other station.

Our goal is to determine a dynamic rebalancing scheme that minimizes the user dissatisfaction while maintaining the rebalancing cost as low as possible. The dissatisfaction is related to the probability that users experience service failures, i.e., find stations either empty ($m_{v}\left(t\right)=0$) or full ($m_{v}\left(t\right)=M_{v}$).(In this work, we do not distinguish between failures due to empty and full stations, though this aspect will be studied in future work.) We assume that a fleet of vehicles with given capacities is available for the rebalancing procedure and that the operating day is divided into discrete time frames of length $T_{r}$. The rebalancing fleet can be deployed at the beginning of each frame.

The rebalancing problem is made up of two main components.

1. At each time frame *k*, we need to determine the desired state $m_{v}^{☆}\left(kT_{r}\right)$ for each station v∈V. Since we would like to maximize the time until a station becomes either empty or full, $m_{v}^{☆}\left(kT_{r}\right)$ is the inventory level that maximizes the *survival time* of the station. This is computed by analyzing the historical user data.
2. The real rebalancing problem, which consists of identifying which stations are either overcrowded or in shortage of bikes, according to their survival times, and designing the truck routes to perform the bikes pickups and deliveries.

In the following, we first describe the procedure to determine the survival times based on the historical data, and then the network-wide rebalancing optimization.

### 3.1. Survival Time

Modeling the network as a whole is not computationally tractable for a large number of stations. However, we can model each station’s occupancy separately as a Markov-Modulated Poisson Process (MMPP) [49]: the occupancy follows a finite Markov BDP $m_{v}\left(t\right)$, where the birth and death processes are Poisson distributed with time-varying rates $\lambda_{v}\left(t\right)$ and $\mu_{v}\left(t\right)$, respectively. We can reasonably assume that the demand for bikes is not affected by the current occupancy of the station (unless it is either empty or completely full(The censoring problem will be described in Section 4.1), and therefore the birth and death rates are assumed to be independent of the current state. The BDP is limited by the station’s capacity $M_{v}$.

In order to reduce the computational complexity [50,51] and accurately estimate $\lambda_{v}\left(t\right)$ and $\mu_{v}\left(t\right)$ from the historical data, we simplify the station behavior as a discrete-time BDP, with birth and death rates that are piecewise constant during time frames of duration $T_{r}$, which is an integer multiple of the BDP slot $T_{p}$. Basically, we map the continuos time *t* into its discrete counterpart

(1) $$t\;\to \;nT_{r}+kT_{p}\;\mathrm{with}\;n=\left\lfloor t/T_{r}\right\rfloor ,\;k=\left\lfloor \left(t-nT_{r}\right)/T_{p}\right\rfloor ,$$

where *n* is the index of the *frame* (which has duration $T_{r}$) and *k* is the index of the *slot* (which has duration $T_{p}$). The two assumptions we have made (piecewise constant birth and death rates, and discrete time) make it possible to have a simpler yet accurate model of the stations’ dynamics, and also to derive the correct parameters of the arrival and departure processes for the available dataset. Once $T_{r}$ was defined, we verified that the empirical distribution of the inter-arrival times in a single time frame $T_{r}$ fits an exponential distribution, thus validating the short-term Poisson assumption.

As mentioned, a service failure occurs whenever a station is in state 0 or in state $M_{v}$: if the stalls are all empty, bikes cannot be rented, while if they are all full, users cannot deposit more bikes. In order to compute the first passage time to either of the two failure states, we can consider an equivalent process in which 0 and $M_{v}$ are absorbing states. Accordingly, we formally define the survival time $S_{v}\left(t,m\right)$ of node *v* as the smallest time τ after which the probability $P_{m,a}\left(\tau ;t\right)$ of reaching state $a\in \{0,M_{v}\}$, starting from state $m\in M_{v}$ at time *t*, exceeds a predefined threshold $p_{\mathrm{th}}$, i.e.:

(2) $$S_{v}\left(t,m\right)=\inf\left\{\tau :P_{m,0}\left(\tau ;t\right)+P_{m,M_{v}}\left(\tau ;t\right)\geq p_{\mathrm{th}}\right\}.$$

Note that $S_{v}\left(t,m\right)$ is time varying, reflecting the fluctuations of the arrival and departure rates. For ease of writing, however, the parameter *t* will be omitted in the following, whenever possible. The threshold $p_{\mathrm{th}}$ makes it possible to tune the reactivity of the rebalancing system: lower values of $p_{\mathrm{th}}$ will yield shorter survival times and, in turn, more frequent rebalancing, which will reduce the service failure risk but increase the operational costs of the service.

Since the birth and death rates vary from frame to frame, the transition probability $P_{i,j}\left(t\right)$ needs to be calculated by considering the state distribution at the end of the previous frame. Applying the Markov property, we get:

(3) $$P_{i,j}\left(t\right)=P_{i,j}\left(nT_{r}+kT_{p}\right)=\sum_{l\in M_{v}}P_{i,l}\left(nT_{r}\right)\;P_{l,j}\left(kT_{p}\right),\;i,j\in M_{v}\;,$$

where *n* and *k* are given by the time relation (1). The transition matrix after $k<T_{r}/T_{p}$ steps is the (k+1)-th power of the one-step transition matrix (note that the time slot index *k* starts from 0 according to the time relation (1)), and then we use the Markov property as in Equation (3) to derive the transition probabilities when the system is in frame *n* and slot *k*.

To compute $P_{m,0}\left(t\right)$ and $P_{m,M_{v}}\left(t\right)$ for a given station *v*, we need to evaluate the transition probabilities starting from state *m* at every time step. In the following, we thus focus only on a single slot of length $T_{p}$, with birth and death rates denoted as $\mu_{v}$ and $\lambda_{v}$, respectively. Let $D_{v}$ and $A_{v}$ be the random variables that describe the numbers of departures and arrivals at station *v* in the considered slot, respectively. We model the demand for bikes (i.e., potential departures) and empty stalls (i.e., potential arrivals) as Poisson variables, with mean $\mu_{v}T_{p}$ and $\lambda_{v}T_{p}$, respectively. We assume that a sequence of events results in the same state regardless of the order of occurrence, so that the next state of the station is given only by the total number of departures and arrivals in a time slot. This is not strictly correct, as different orderings of the same events might bring the system in an absorbing state. In general, however, the probability of this event is sufficiently low and does not significantly affect the accuracy of the simplified model. The probability $P_{i,j}$ of going from state *i* to state *j* in one time step $T_{p}$ is

(4) $$\begin{array}{cc} P_{i,j} & =\mathrm{Pr}\left[A_{v}-D_{v}=j-i\right]=\sum_{\ell =-\infty }^{\infty }\mathrm{Pr}\left[A_{v}=j-i+\ell \right]\mathrm{Pr}\left[D_{v}=\ell \right]\\ & =\sum_{\ell =\max\{0,j-i\}}^{+\infty }\frac{{\left(\lambda_{v}T_{p}\right)}^{-(j-i+\ell )}\;{\left(\mu_{v}T_{p}\right)}^{-\ell }}{\ell !\;(j-i+\ell )!}\;, \end{array}$$

where the last equality follows from the Poisson distribution of $A_{v}$ and $D_{v}$. The result of the sum in Equation (4) follows a truncated Skellam distribution [52]:

(5) $$p_{\mathrm{Sk}}\left(\ell ;\mu_{1},\mu_{2}\right)=e^{-(\mu_{1}+\mu_{2})}{\left(\frac{\mu_{1}}{\mu_{2}}\right)}^{l/2}I_{\left|\ell \right|}\left(2\sqrt{\mu_{1}\mu_{2}}\right)\;,$$

with ℓ=j−i, $\mu_{1}=\lambda_{v}T_{p}$ (the average of $A_{v}$), and $\mu_{2}=\mu_{v}\left(t\right)T_{p}$ (the average of $D_{v}$). $I_{k}\left(z\right)$ is the modified Bessel function of the first kind and order *k* [53]:

(6) $$\begin{array}{cc} I_{\alpha }\left(x\right) & =\lim_{N\to \infty }\sum_{n=0}^{N}\frac{1}{n!\Gamma (n+\alpha +1)}{\left(\frac{x}{2}\right)}^{2n+\alpha }\;, \end{array}$$

(7) $$\begin{array}{cc} \Gamma (z) & =\int_{0}^{\infty }x^{z-1}e^{-x}dx\;. \end{array}$$

The mass probability function given in Equation (5) is for the standard Skellam distribution, while we need to truncate it over the finite range $\left\{0,\cdots ,M_{v}\right\}$. Thus, the transitions to the two absorbing states 0 and $M_{v}$ have the following probabilities:

(8) $$\begin{array}{cc} P_{i,0} & =\sum_{k=-\infty }^{0}p_{\mathrm{Sk}}\left(k-i;\mu_{1},\mu_{2}\right)\;,\;\;i\in M_{v}\backslash \left\{0,M_{v}\right\}\;, \end{array}$$

(9) $$\begin{array}{cc} P_{i,M_{v}} & =\sum_{k=M_{v}}^{+\infty }p_{\mathrm{Sk}}\left(k-i;\mu_{1},\mu_{2}\right)\;,\;\;\;i\in M_{v}\backslash \left\{0,M_{v}\right\}\;. \end{array}$$

Since 0 and $M_{v}$ are absorbing states, $P_{0,0}=P_{M_{v},M_{v}}=1$, while $P_{0,j}=0\;\forall j\neq 0$ and $P_{M_{v},j}=0\;\forall j\neq M_{v}$.

In practice, the transition probabilities at time instant *t* are computed in three steps with a time hierarchical interrelation, which exploits the time equivalence of Equation (1):

1. We use the Markov property to separate the problem at a frame level; to calculate the transition probabilities in frame n>0, it is necessary to compute those at the end of each frame $n^{\prime }<n$.
2. Within a single frame *n*, the transition probabilities at slot k>0 are derived by elevating the single-step transition matrix of frame *n* to the power of k+1 (since slot indices start from 0).
3. The transition probabilities at the beginning of a frame (i.e., for slot index k=0) are then computed using Equations (4), (8) and (9).

Hence, for each time instant *t* and starting state *m*, we are able to compute the probability of being in one of the failure states, 0 or $M_{v}$, and, consequently, the survival time $S_{v}\left(t,m\right)$ of station *v*. Intuitively, $S_{v}\left(t,m\right)$ is first increasing and then decreasing in *m* (it is 0 for both m≡0 and $m\equiv M_{v}$), and there exists a state $m_{v}^{☆}\left(t\right)$ that leads to the maximum survival time $S_{v}^{☆}\left(t\right)$:

(10) $$m_{v}^{☆}\left(t\right)=\underset{m\in M_{v}}{\mathrm{argmax}}S_{v}\left(t,m\right)\;\mathrm{and}\;S_{v}^{☆}\left(t\right)=S_{v}\left(t,m_{v}^{☆}\left(t\right)\right),\;v\in V.$$

Ideally, at time *t*, we would like to be in state $m_{v}^{☆}\left(t\right)$ as it corresponds to the longest time for which station *v* is self-sufficient (i.e., it does not need rebalancing). Next, we describe how to plan the rebalancing operation by exploiting the knowledge of the survival times.

### 3.2. Network-Wide Optimization

We model the dynamic rebalancing problem as an optimization problem on the bike-sharing network graph G. The number of deployed trucks is predefined and denoted as *X*, and we assume that the subsets of stations visited by each vehicle are disjoint. Each truck has a given capacity, which defines the maximum number of bikes it can transport concurrently. Then, we define a circular graph $H=(V^{\prime },F)$, where $V^{\prime }\subseteq V\cup \left\{0\right\}$ is the union of the set of stations that the fleet of trucks rebalances (⊆V) and the truck depot ({0}), while F is the set of edges that solves the Vehicle Routing Problem (VRP) [54] on $\left(V^{\prime },E^{2}\right)$.

We denote the station occupancy vector before rebalancing as $m^{\left(⌀\right)}\left(t\right)≜\left[m_{1}^{\left(⌀\right)}\left(t\right),\cdots ,m_{\left|V\right|}^{\left(⌀\right)}\left(t\right)\right]$, while the occupancy vector after rebalancing is $m^{\left(H\right)}\left(t\right)$. The obtained reward is measured as the time gained before a system failure occurs, i.e., the difference between the minimum survival times among all the stations after and before the rebalancing operation. Each rebalancing operation also implies a fixed cost for each vehicle in the fleet, plus an additional cost that depends on the total length $D_{x}\left(H\right)$ of the route of each vehicle x∈[1,⋯,X]. The rebalancing optimization function $\tilde{f}\left(H,t\right)$ can thus be modeled as

(11) $$\tilde{f}\left(H,t\right)=\left(\min_{v\in V}S_{v}\left(t,m_{v}^{\left(H\right)}\left(t\right)\right)-\min_{v\in V}S_{v}\left(t,m_{v}^{\left(⌀\right)}\left(t\right)\right)\right)-\alpha X-\beta \sum_{x=1}^{X}D_{x}\left(H\right)\;,$$

where and α≥0 and β≥0 are two weights (measured in time units and time units per kilometer, respectively), and the survival times $S_{v}\left(\cdot ,\cdot \right)$ are computed as described in Section 3.1.

We introduce a further parameter γ in the optimization function, which indicates the clipping threshold for the survival time: this has the dual purpose of disregarding longer survival times, which might have a larger estimation error, and avoiding useless rebalancing operations when the minimum survival time of the system is already very long. The optimization function then becomes

(12) $$f\left(H,t\right)=\left(\min\left\{\min_{v\in V}S_{v}\left(t,m_{v}^{\left(H\right)}\left(t\right)\right),\gamma \right\}-\min\left\{\min_{v\in V}S_{v}\left(t,m_{v}^{\left(⌀\right)}\left(t\right)\right),\gamma \right\}\right)-\alpha X-\beta \sum_{x=1}^{X}D_{x}\left(H\right).$$

As discussed in the introduction of Section 3, the operating day is divided into discrete frames of length $T_{r}$. The rebalancing problem is solved periodically, every $T_{r}$, and consists of defining $V^{\prime }$ and F, which means i) identifying which stations require rebalancing, and ii) determining the path for the rebalancing truck. Note that, if at time *t* the optimization function f(H,t) is negative for any $V^{\prime }\neq 0$, the cost of moving the vehicle fleet is larger than the reward obtained for reallocating the bikes, and thus no rebalancing is performed. In this case, $V^{\prime }=\left\{0\right\}$.

Since the optimization function also depends on the distance covered by the rebalancing truck, the set of stations to visit, $V^{\prime }$, and the vehicle route, F, should be jointly determined. For simplicity, we first assume to know the minimum path length of the rebalancing route for a given set of nodes, so that we can derive $V^{\prime }$ and then describe how to compute such distance. The computation is simplified by the following theorem, which states that $V^{\prime }$ contains the nodes with the smallest survival times, so that all subsets that do not satisfy the theorem can be safely discarded as suboptimal.

**Theorem** **1.** If $v\in V^{\prime }$, then $S_{v}\left(t,m_{v}^{\left(⌀\right)}\left(t\right)\right)<S_{u}\left(t,m_{u}^{\left(⌀\right)}\left(t\right)\right)\;\forall u\in V\backslash V^{\prime }$

**Proof.**  Assuming the truck follows the optimal route F, adding a node to H cannot decrease the distance $D_{x}\left(H\right)$ because of the triangle inequality. It follows that, in order for *v* to be part of the rebalancing set $V^{\prime }$, its presence needs to increase the first term of the function in (12), i.e., $S_{v}\left(t,m_{v}^{\left(⌀\right)}\left(t\right)\right)<\min_{u\in (V\backslash V^{\prime })}S_{u}\left(t,m_{u}^{\left(⌀\right)}\left(t\right)\right)$. ☐

The procedure to determine $V^{\prime }$ is hence described in Algorithm 1. Intially, $V^{\prime }$ only contains the deposit node 0 (Line 1); then, at each iteration *i*, we identify the node $v\left(i\right)\in V\backslash V^{\prime }$ with the minimum survival time (Line 9). We compute the reward obtained by adding v(i) to $V^{\prime }$, as if its survival time were optimal, $S_{v(i)}\left(t\right)\equiv S_{v(i)}^{☆}\left(t\right)$ (Line 10), and the cost to add it to the truck route (Line 14). In particular, the reward is computed as the difference between the smallest survival time in set $V^{\prime }$ and the smallest survival time before rebalancing (Lines 4 and 11), while the cost depends on the new route length (see Equation (12)). If the optimization function increases (Line 15), node v(i) is indeed added to $V^{\prime }$. The algorithm stops if either all nodes of V have been included in $V^{\prime }$, or the optimization function can no longer be increased because the minimum of the optimal survival times among all visited nodes $w\in V^{\prime }$ after rebalancing is smaller than the current survival time of any of the unvisited nodes (those in $V\backslash V^{\prime }$) (Line 18).

The solution of the dynamic rebalancing problem has two distinct parts: (i) determining the route for the set of stations that need to be rebalanced (Line 12 in Algorithm 1) including possible additional constraints given by vehicle capacity and travel times, and (ii) deciding whether to start the rebalancing operation and, possibly, which stations to visit. The literature on the first part of the problem is already extremely vast and well-developed. In particular, any of the solutions discussed in Section 2.2 can be used to determine the rebalancing path. Instead, there are very few studies on the second problem, i.e., deciding which station to rebalance and when, which is the focus of our model. We remark that the path-planning algorithm has an impact on the rebalancing strategy provided by our approach, since the cost computed in Line 14 includes the covered distance that, in turn, depends on the chosen path. However, our framework is general and can accomodate any path-planning strategy.

**Algorithm 1** Rebalancing strategy1:Initialize i=0, $V^{\prime }=\left\{0\right\}$, f(H,t)=0, done =02:Set parameters α,β,γ,X
3:Set $S\left(t\right)=[S_{1}\left(t,m_{1}^{\left(⌀\right)}\right),\cdots ,S_{\left|V\right|}\left(t,m_{\left|V|\right|}^{\left(⌀\right)}\right)]$▹ Vector with survival times before rebalancing4:Set $\sigma =\min_{w\in V}S\left(t\right)$▹ Smallest survival time before rebalancing5:σ=min{σ,γ}▹ Set the survival time threshold6:Set $S^{☆}\left(t\right)=\left[S_{1}^{☆}\left(t\right),\cdots ,S_{\left|V\right|}^{☆}\left(t\right)\right]$▹ Vector with optimal survival times for each station v∈V7:**while** (i<V) and (done ==0) **do**▹ Until there are nodes to visit that can improve the net reward8: i←i+1
9: $v\left(i\right)\leftarrow {\mathrm{argmin}}_{w\in V\backslash V^{\prime }}S\left(t\right)$▹ Choose unvisited node with smallest survival time10: ${\left[S\left(t\right)\right]}_{v(i)}\leftarrow {\left[S^{☆}\right]}_{v(i)}\left(t\right)$▹ Update survival time of node v(i)11: reward←min{minS(t),γ}−σ▹ Update reward12: Determine rebalancing path F over $V^{\prime }\cup v\left(i\right)$
13: D← compute path length of F▹ Compute distance to cover for rebalancing14: cost←αX+βD▹ Update cost15: **if** (reward − cost) >f(H,t)
**then**▹ It is worth to include node v(i) in the rebalancing16:  $V^{\prime }\leftarrow V^{\prime }\cup v\left(i\right)$17:  f(H,t)← reward − cost18: **if**
$\min_{w\in V^{\prime }}\left\{{\left[S^{☆}\left(t\right)\right]}_{w}\right\}<\min_{w\in V\backslash V^{\prime }}\left\{{\left[S\left(t\right)\right]}_{w}\right\}$
**then**19:  done ←1▹ The optimization function f(H,t) can no longer be increased

The path-planning problem can be *optimally* solved by the well-known VRP, which yields the lowest possible cost. However, the VRP is an NP-hard problem, so that several efficient optimization algorithms have been defined in the literature for different scenarios and sets of assumptions, in order to reduce the computational complexity. In our simulation, we made the following assumptions to simplify the problem:

1. We use the Euclidean distance between stations as distance metric for the set of edges $E^{2}$. This implies that $d\left(v_{i},v_{j}\right)\equiv d\left(v_{j},v_{i}\right)$ for any two stations *i* and *j*.
2. We consider a single rebalancing vehicle, i.e., X=1, with infinite capacity. Notice that this scenario reduces the VRP to the Traveling Salesman problem.

The Traveling Salesman problem is still NP-hard. Since the focus of this work is on the dynamic algorithm and not on the solution to the routing problem, we opted for a simple heuristic that greedily chooses the closest unvisited node at each step. Let $U_{i}\subseteq V^{\prime }$ be the set of nodes visited up to iteration *i*. Since the truck deposit is the first node to be visited, we set $U_{0}=u\left(0\right)\equiv \left\{0\right\}$, while for $i\in \{1,\cdots ,|V^{\prime }|-1\}$ (Note that the last iteration is $i=|V^{\prime }|-1$ because $V^{\prime }$ includes also the deposit node {0}.), we have $U_{i}=U_{i-1}\cup \left\{u_{i}\right\}$, where $u_{i}$ is the node visited at iteration *i*, such that

(13) $$u_{i}=\underset{w\in V^{\prime }\backslash U_{i-1}}{\mathrm{argmin}}d\left(w,u_{i-1}\right)\;,$$

where, as mentioned, d(x,y) is the Euclidean distance between nodes *x* and *y*. This rule implements the well-known *nearest-neighbor* heuristic, which has often been used as a benchmark for Traveling Salesman heuristic solutions [55]. The total path distance covered by the rebalancing truck is

(14) $$D\left(H\right)=\sum_{i=1}^{|V^{\prime }|-1}d\left(u_{i},u_{i-1}\right)+d\left(u_{|U|-1},0\right),$$

where the last term is the distance the vehicle covers to go back to the deposit after the rebalancing operations. This heuristic can be computed in $O(|V^{\prime }{|}^{2})$ time. The total running time of the heuristic algorithm is $O(|V{|}^{3})$, since $|V^{\prime }\left|\leq |V\right|$. Although using a greedy heuristic may not give the optimal routing solution, solving an NP-hard problem might not be practical for large instances.

## 4. Data Analysis

**Figure 1:**
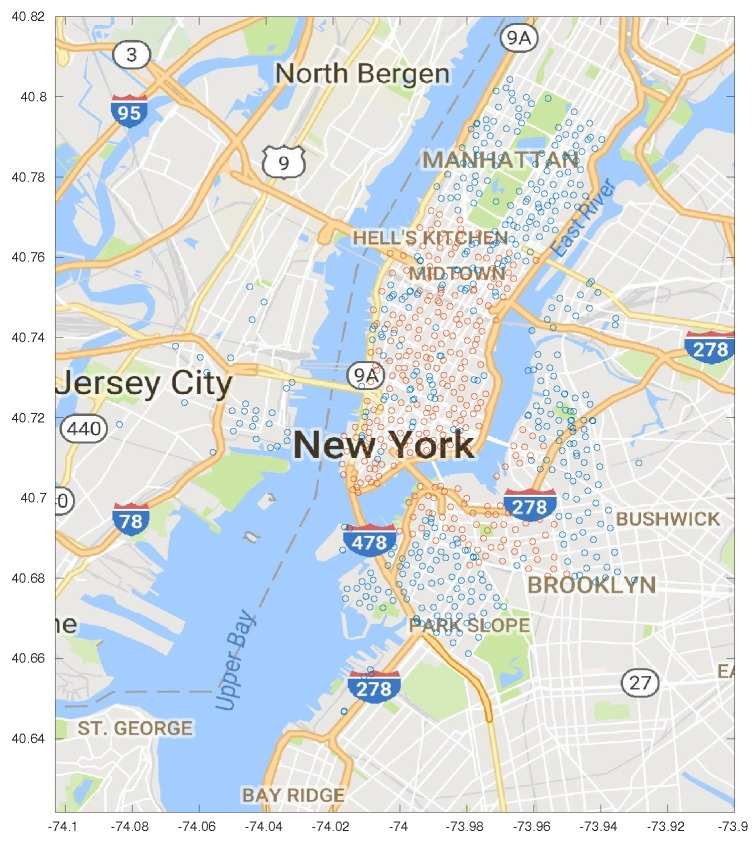
Map of the bike-sharing stations: red points identify the stations considered in our study.

In this work, we used the publicly available dataset (https://www.citibikenyc.com/system-data) from New York City’s CitiBike network, which we briefly described in Section 1. A map of the city, with the positions of the docking stations, is shown in Figure 1.

The service publishes monthly reports with all the recorded rides, the start and destination times and stations, the unique identifiers of the bikes, and some records about the service subscribers, such as year of birth and gender. In this study, we use the data from July 2013 (the earliest available period) to July 2017. Since several stations were added during the considered time frame, we limit ourselves to the 280 stations that were present for a sufficiently long time to extract the demand data. (By discarding some of the stations, the system is not closed; bikes enter and exit it as they travel to other stations. We clarify how we deal with this issue in Section 5.)

### Traffic Analysis and Simulation

**Figure 2:**
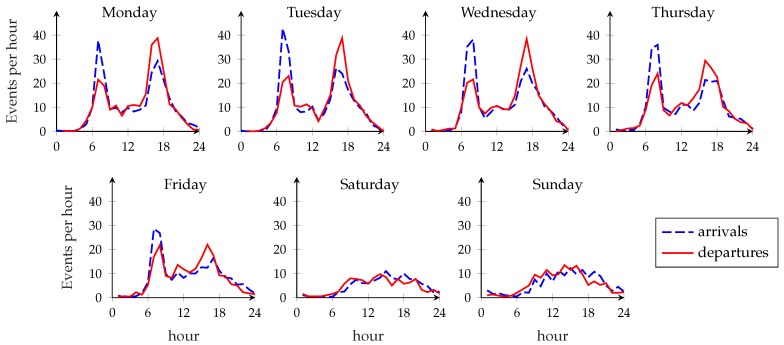
Traffic patterns for the month of July 2015 at station 537 (Lexington Ave. and East 24th St.)

The traffic pattern intuitively depends on several factors, such as the time of the year (fewer people tend to use bikes in winter than in summer), whether it is a weekday or weekend (people may tend to use bikes at more regular times on weekdays and for shorter periods), and the position of the considered station (and the facilities nearby), as shown in Figure 2, which reports the traffic pattern for a station close to CUNY Baruch College during the month of July 2015. On weekdays, the rush hours around 8 a.m. and 5 p.m. can be identified by the spikes in the demand for bikes; since the station is probably used by many students and workers, there are more arrivals than departures in the morning and more departures than arrivals in the evening. On Fridays, there is a more homogeneous pattern in the afternoon, likely because many people leave work early, and the demand is much lower during the weekend. These patterns highlight that both the day of the week and the hour need to be considered when calculating the arrival and departure rates. Another important factor is the weather: the summer months have the highest demand for bikes, while the service is used far less during colder months.

This fits with the simplifying assumptions in Section 3. We assume that the Poisson rates $\lambda_{v}\left(\cdot \right)$ and $\mu_{v}\left(\cdot \right)$ for the arrival and departure process, respectively, are piecewise constant at steps of duration $T_{r}$ equal to one hour. Hence, we can extract the value of the arrival and departure rates for each period Ω, defined as the hour, day, and month of the year, for all the stations in the network. Since the dataset contains four years of data, we calculate $\lambda_{v}\left(\Omega \right)$ and $\mu_{v}\left(\Omega \right),\;v\in V$ as the mean arrival rate in the first three years, from July 2013 to June 2016. The test set on which we perform the optimization is the last year of data, from July 2016 to June 2017. This provides a sizeable amount of data, though the increasing of the number of stations and subscribers affects the accuracy of the estimates, which is unavoidable when using past data for future optimization.

There are some other issues that need to be mentioned. Stations that are empty or full prevent customers from taking or returning bikes, so that the observed trip data may actually differ from the true demand and rather represent a lower bound for it. In addition, the bike demand may increase thanks to the rebalancing operations, as people would see that the system is becoming more efficient. Unfortunately, we have no way to gauge the real demand from the available data, and the effect of the current rebalancing operations prevents the extrapolation of the real state of the system at any given time. However, the framework we propose is general and can be easily adapted to different datasets. Thus, although the numerical evaluation could be affected by the censoring issue, the model and the proposed solution have general validity.

## 5. Results

In order to validate our optimization scheme, we compare its performance with two other scenarios: a system with no rebalancing and one with static rebalancing. In the first case, we just let the bike-sharing system evolve without intervening. In the second one, we bring the whole network to the optimal state twice a day, at 3 a.m. and 3 p.m., as in [8]. In our simulations, we make the following assumptions.

- The CitiBike data accurately represent the demand for bikes. As discussed in Section 4.1, this may not be strictly true because of the censoring effect, so that the demand patterns in the data represent a lower bound for the real demand.
- Bikes coming from or going to stations that are not considered in the optimization framework because of lack of data are assumed to be new bikes entering the system, or broken bikes exiting it, respectively.
- If a user does not find a bike at their desired station, they simply exit the system and the trip does not happen. If they find a bike at their departure station but do not find a free spot at the arrival station, the bike exits the system.
- At the beginning of every month, the state of each station is reset to the optimal value. Since the dataset does not provide the state of each station, this assumption was necessary to define the initial state of the system.

Naturally, the optimization schemes themselves are not affected by these assumptions, but the numerical results of the simulation may change when choosing different assumptions. The simulations were run for a whole year of data, from July 2016 to June 2017, and the results are presented on a month-by-month basis. We set the cost of taking a rebalancing trip, α, to 2700 s (45 min); since the duration of a slot $T_{p}$ is 15 min, this means that the minimum survival time must improve by at least an hour for the system to consider a rebalancing operation. The parameter β, which represents the cost of moving the rebalancing truck, was set to four different values, namely 0.02, 0.04, 0.08, and 0.12 s/m.

We used the data without any optimization scheme to test the assumptions of our model. First, we verified the accuracy of the Poisson model for the interarrival times by considering the data of several randomly chosen stations, which exhibited an extremely good fit with the exponential distribution, despite the relatively small sample.

The second test was intended to check the validity of the survival time formula, for which we run the prediction without implementing any rebalancing strategy, in order not to perturb the system evolution. This test was performed for two randomly chosen weeks in September 2016 and January 2017, and the results were extremely good, since the empirical distribution of the survival time obtained from the dataset was almost overlapping with our model, with a relative error on the survival time lower than 1.5%. The slight discrepancy can be explained by time-dependent factors such as the yearly increase in the demand for bikes, which causes the model to slightly underestimate the demand in late 2016 and early 2017 since we computed the survival time using the data from July 2013 to June 2016.

**Figure 3:**
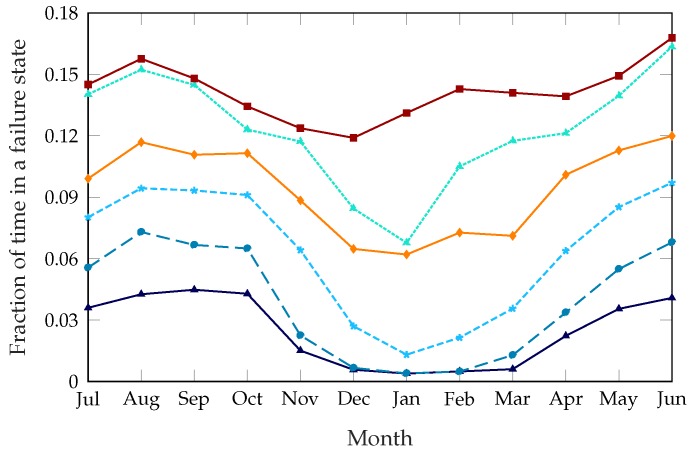
Monthly average probability of being in a failure state, over a year.

***Service improvement.*** Figure 3 shows the fraction of time that the bike-sharing system spends in a failure state, i.e., either completely full or empty, averaged for each station and for each month of the year. We used this metric instead of the number of service failures because it is less affected by the censoring problem we described in Section 4.1, as requests that could not be fulfilled are not shown and would not be counted in the results.

The unoptimized system has a failure probability of about 14%, which means that, without any rebalancing intervention, stations cannot satisfy the users’ needs for about 14% of the time. The static rebalancing strategy from [8], which optimizes the whole system twice a day at preset times, can improve this to about 11% during the summer and 6% during the winter. This discrepancy is due to the far lower demand during colder months, which makes rebalancing actions more effective. The dynamic system can improve the service even further: with low values of β, the failure time can be as low as 3% during the summer and 0.4% during the winter. This obviously comes at a cost, as an aggressive rebalancing strategy will result in more daily trips and a higher cost to move the trucks. However, the framework we propose makes it possible to tune the dynamic scheme to the specific requirements of the system, in order to balance the operational costs and quality of the service as desired. For example, setting β=0.12, we can approximately replicate the performance of the static scheme, as shown in Figure 3.

**Figure 4:**
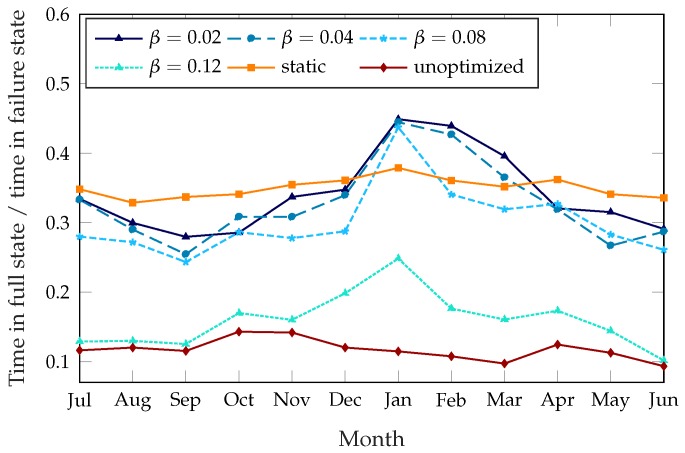
Fraction of failures due to full stations, over a year.

In order to have a full picture of the effectiveness of the scheme, we need to consider not only the total failure time but also the kinds of failures, as the two failure modes have a different impact on the behavior of the users and on the future planning of the system. Figure 4 shows the ratio of the time the stations spend completely full over the total failure time. The unoptimized system has a very skewed failure mode, with almost 90% of failures due to empty stations. The static rebalancing scheme has a full station ratio of about 35%, which is almost constant throughout the year. The dynamic rebalancing schemes show a higher ratio of full stations during the winter months, mirroring the overall pattern from Figure 3: since most of the failures during the summer months are due to empty stations, the improvement during the winter comes from the stations being empty less often, while the time they spend full does not decrease as much. This effect is particularly significant for β=0.12, which shows a variation from 15% in summer to over 30% in winter, while the other settings of the dynamic scheme are more similar to the static case.

***Rebalancing costs.*** The improvement in the service obviously comes at a cost: the fleet of rebalancing trucks must be deployed several times a day, and a more aggressive rebalancing strategy increases both the number of trips and the total distance that needs to be covered, with higher fuel and personnel costs.

**Figure 5:**
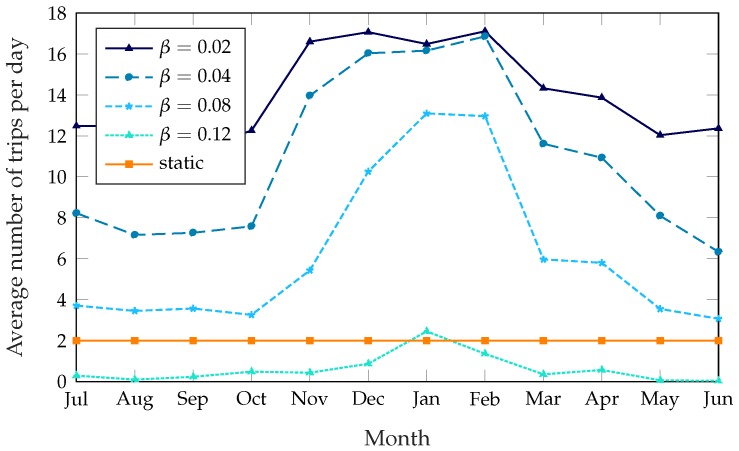
Average number of rebalancing trips per day, over a year.

Figure 5 shows the number of trips the rebalancing truck has to take every day, averaged over each month; the unoptimized system is not shown in this figure, since no trips are taken at all. The static scheme has a constant number of trips per day, since it explicitly requires the truck to take two trips at predetermined times. As for the dynamic schemes, the large difference between the summer and winter months might be counterintuitive, as one would expect a lower demand to correspond to a lower number of trips. However, when demand is lower survival times are larger in general; stations that are not in their optimal state might therefore benefit more from a rebalancing, increasing the potential reward. Since the cost of rebalancing the system is constant, rebalancing the system is often more rewarding during the winter, resulting in a higher number of trips and a better service, as Figure 3 and Figure 5 show. This issue is exacerbated by time-dependent factors: as described in Section 5, most of the anomaly disappears if we use the real demand data instead of the estimate based on historical patterns, and this can be explained by weather patterns, as 2017 was a particularly warm and dry winter.

**Figure 6:**
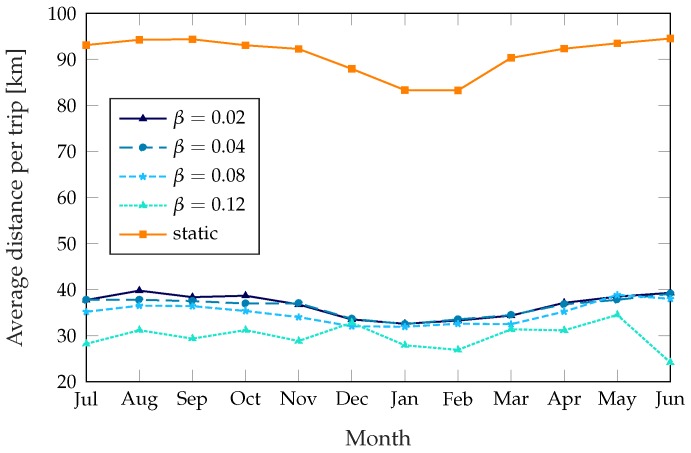
Average distance per rebalancing trip, plotted over 12 months.

**Figure 7:**
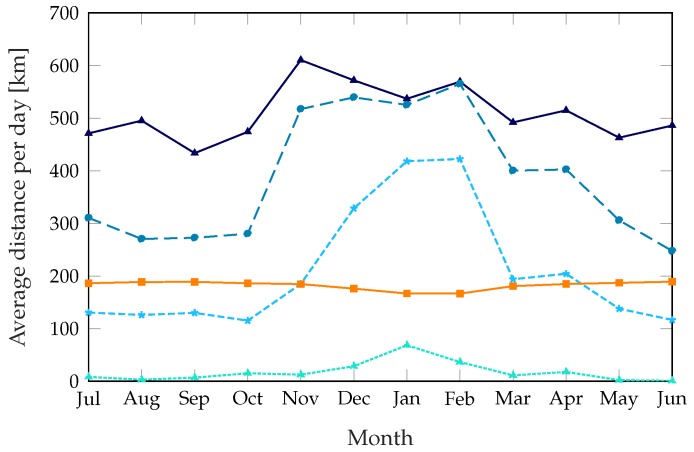
Average distance covered by rebalancing trucks per day, plotted over 12 months.

The dynamic schemes need a higher number of trips to get the performance improvement, but the distance the trucks cover on each trip is far lower: while the static scheme rebalances the whole system and covers every station that needs rebalancing, the dynamic schemes only target the stations that need it most. As Figure 6 shows, the dynamic schemes need between 30 and 40 km per trip, while the static scheme can need more than 90 km during the summer. Figure 7 shows the average distance the truck covers during a day: even if the dynamic scheme with β=0.04 takes more than twice as many trips as the static scheme, with a corresponding performance benefit, the total distance it covers is similar during the summer. The scheme with β=0.12 reaches a service quality similar to the static scheme’s while taking fewer trips in the summer and covering a smaller distance during most of the year. The parameter α, which we kept at a constant value of 2500 s throughout these simulations, can be used to change the preference of the system from frequent short trips to fewer and longer ones.

In general, the dynamic schemes can take shorter trips, potentially deploying a smaller fleet. (We remark that we only consider one rebalancing truck in this work; future developments are going to address this issue, as the model and optimization function we propose are fully general.) The dynamic nature of our solution allows us to tune the rebalancing to the needs of the system, giving system controllers the freedom to choose the optimal point in the trade-off between service quality and rebalancing costs by changing α and β.

**Figure 8:**
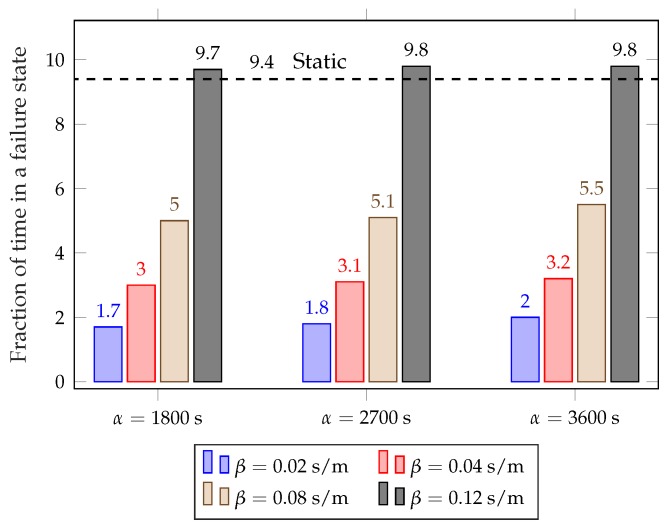
Average fraction of time in a failure state (completely empty or full station) for different values of α and β.

**Figure 9:**
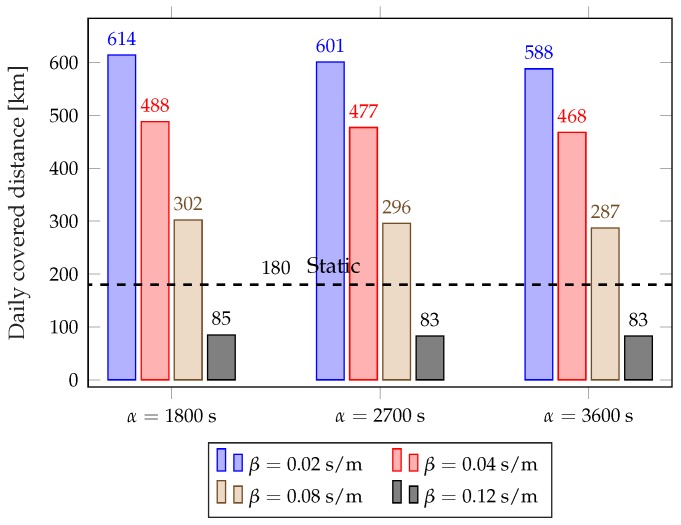
Average daily distance covered by rebalancing trucks for different values of α and β.

Figure 8 and Figure 9 show the average failure rate and average daily distance covered by the rebalancing trucks for different values of α and β, considering the full year. The plots clearly show the trade-off between rebalancing costs and service performance: using lower values of β results in a lower failure rate, but the rebalancing trucks need to be deployed more often and, consequently, cover far more distance every day. The dynamic scheme with β=0.12 results in a slightly higher failure rate than the static scheme, but requires less than half the rebalancing distance. The parameter β is the key to choose the operating point in the trade-off, as it has a significant impact on the performance of the scheme. The value of α has a limited effect over the considered range, so it can be used for fine-tuning the system after β has been selected.

**Figure 10:**
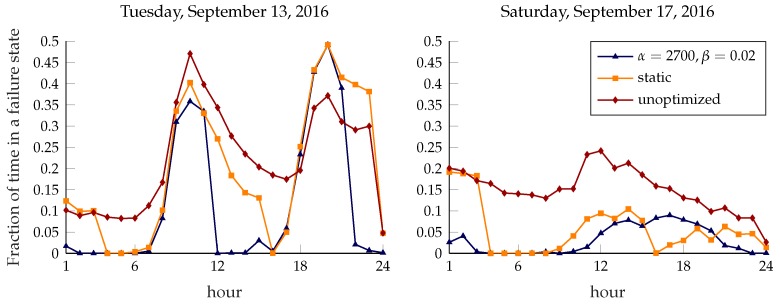
Average fraction of time in a failure state, plotted over the course of the day

***Evolution of the system over time.*** Figure 10 shows the average fraction of time that a station spends in a failure state for 13 September (Tuesday) and 17 September (Saturday) 2016. As shown in Figure 2, the difference between a weekday and a holiday is striking: in the first plot, the two intense demand periods starting around 7 a.m. and 5 p.m. are evident from the steep increase of the failure rate, which is almost entirely absent in the other one. The limitations of the static approach to rebalancing can be clearly seen in the first plot: while the rebalancing operation at 3 p.m. brings the failure rate very close to 0 in the following hour, the system is left to itself during rush hour, and the failure rate quickly rises to match that of the unoptimized system. Even though the dynamic approach also has a peak failure rate at rush hour, it quickly recovers and keeps the system in a better state throughout the day; the bike-sharing system is probably underdimensioned for the peak demand, so it is hard for any rebalancing scheme to avoid failure during rush hour, but the dynamic system still yields a significant improvement. The weekend plot shows a similar pattern for the static approach, with perfect functioning right after rebalancing and a steadily increasing failure rate afterwards. However, the lower and more homogeneous demand makes rebalancing less urgent and decreases the gain of using a dynamic approach.

**Figure 11:**
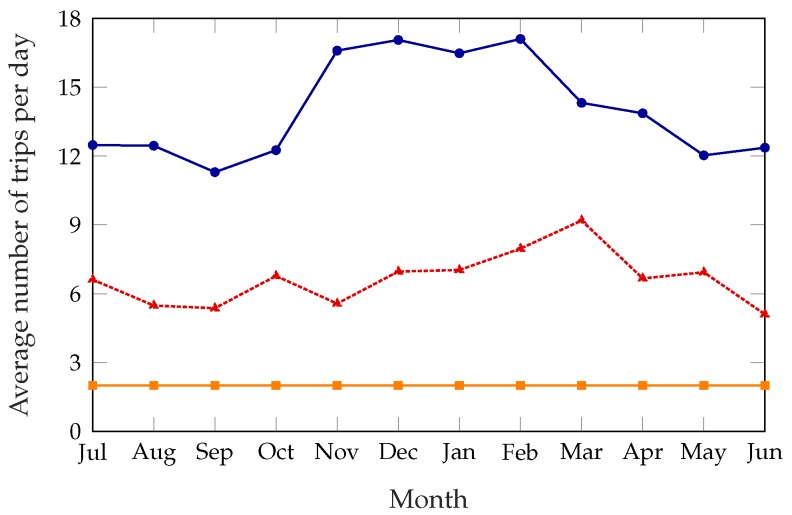
Average number of rebalancing trips per day with perfect and imperfect demand information, plotted over 12 months.

***Effects of the demand estimation error.*** Figure 11 shows the effects of the estimation error in the demand data: we ran the simulations again, giving the system a perfect estimate of the demand for each hour, and used the performance as a benchmark. During the winter months (from November to February), the estimation error leads the system to take far more rebalancing trips (the distance covered on each trip was similar); a quick analysis of historical weather data suggests that the 2016–2017 winter was both considerably warmer and less snowy than previous years; this is the most plausible cause for the difference between the actual demand patterns and the estimates, which relied on data from previous years.

**Figure 12:**
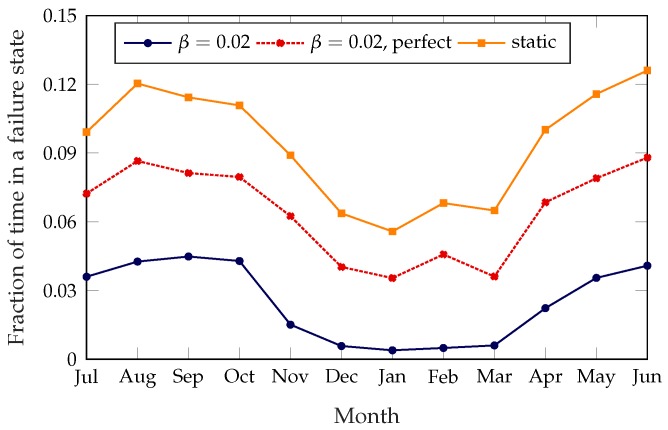
Average fraction of time in a failure state with perfect and imperfect demand information, plotted over 12 months.

Figure 12 shows the effect that estimation errors have on the service quality. The system with perfect knowledge tends to rebalance far less because of its better knowledge of the demand; consequently, its failure rate is slightly higher (although still significantly lower than that of the static approach), but it needs half as many trips. We remind the reader that the trips taken by the dynamic systems, which do not rebalance the whole bike-sharing system at once, are far shorter than those in the static system; as such, the system with perfect knowledge gets a performance improvement over the static system while keeping the same rebalancing costs. A more aggressive setting of the α and β parameters would bring the failure rate closer to that of the imperfect information system, while presumably still limiting the rebalancing costs. An adaptive system that actively corrects the estimates based on a combination of current usage patterns, weather predictions and extraordinary events instead of relying on data from previous years would definitely improve the performance of the system. This is perfectly in line with the Smart City paradigm, which advocates a tighter integration of city services to exploit correlations in the data they generate. The remaining seasonal variations can either be accepted or smoothed out by adapting the parameters α and β to the seasonal behavior patterns of the system.

**Figure 13:**
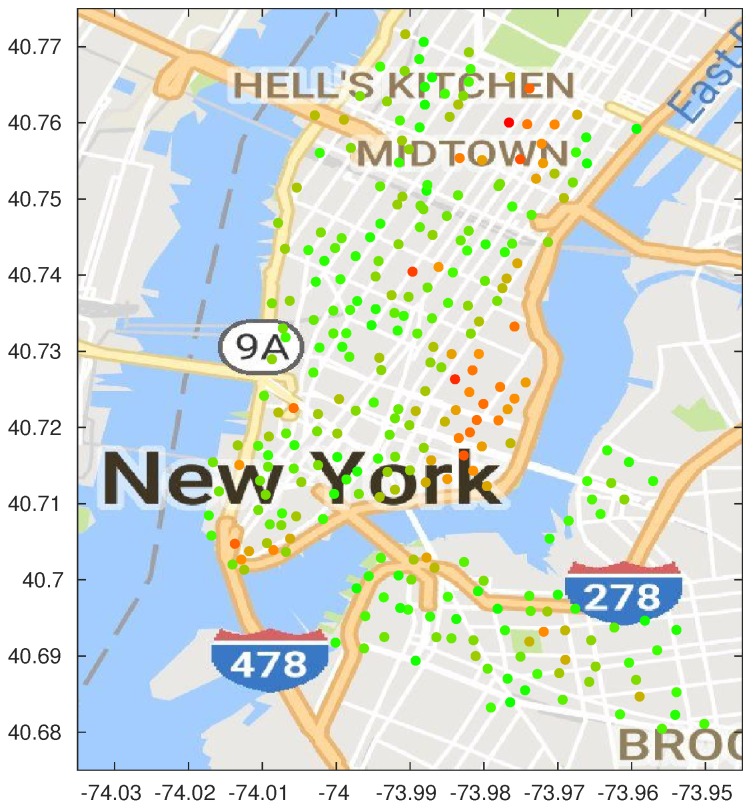
Map of the bike-sharing stations, color-coded from green (zero failure rate) to red (maximum failure rate).

***Critical stations and consequences for system planning***. Figure 13 shows a map of the optimized stations, color-coded based on their failure rate. Green stations have a lower than average failure rate, while the orange ones have an extremely high failure rate. The only red station is the one with the highest failure rate, almost four times the average. The map clearly shows two clusters of congested stations in the Midtown and East Village neighborhoods; although the map was based on the results of the dynamic algorithm with β=0.04, the same pattern can be seen across all the considered schemes.

Although CitiBike is mostly focusing on expanding throughout Upper Manhattan and to the other boroughs, increasing either the number of stations or the capacity of the existing ones in these two congested areas would greatly increase the quality of the offered service. The presence of stations that are often empty or completely full also contributes to further skew the data from the real demand, exacerbating the censoring problem we discussed in Section 4.1. A more thorough analysis of stations’ criticality, including time-dependent patterns and correlations with public transport schedules, would be extremely useful to improve both the bike-sharing and mass transit systems.

## 6. Conclusions

In this paper, we proposed a general framework for the dynamic rebalancing of a bike-sharing system, in order to improve the availability of the service, i.e., to prevent stations from becoming either completely full or completely empty. A periodical scan of the network can identify overcrowded or almost empty stations, which will not be able to support the expected bike demand without human intervention to balance the system. Bike-sharing usage is estimated by analyzing the historical data, which makes it possible to identify traffic patterns over time. In particular, we extrapolated bike arrival and departure rates for each station that depend on the hour, the day of the week, and the month. This information is used to model each bike station through a BDP. In this way, we are able to estimate the time until the station becomes either empty or full with a high probability. Rebalancing is performed if the gain obtained by reallocating bikes exceeds the cost of moving the rebalancing truck. We identify the stations to visit and a greedy rebalancing path.

The results we obtained show that the model we defined is realistic and flexible, and that a dynamic approach to rebalancing can outperform static ones and allow system controllers to decide whether to prioritize maintenance costs or service quality. The dynamic approach can also reduce the rush hour problem during the weekdays, keeping the quality of the service more stable and avoiding high failure rates during times of peak demand. The data from the system can also be used for future planning, as critical stations and areas are easy to identify. Finally, the results show that some of the issues of rebalancing systems are due to an inaccurate estimation of the demand patterns; in order to be more effective, the system should not just take into account historical data, but also current trends and weather data.

We would like to stress that, although we made some simplifications for the sake of mathematical tractability, the model we proposed is of general validity. In particular, the rebalancing optimization function of Section 3.2 can readily accommodate different costs and rewards by appropriately tuning its parameters. Furthermore, although we specifically provided a simple heuristic to compute the rebalancing path for a single vehicle, the rebalancing algorithm (1) considers a fleet with an arbitrary number of trucks. Similarly, we focused on a scenario where the cost between two stations is independent of the link direction and is only defined by the Euclidean distance between the nodes, but our model envisages a directed graph with different costs, so as to model the presence of one-way streets or other differences in the two directed links between two stations. The simplified framework we proposed already improves the bike-sharing system, and this encourages us to relax some assumptions and adopt a more accurate model to obtain further performance enhancements. We plan to consider multiple vehicles in charge of rebalancing bikes, since in big cities it is unrealistic and probably inefficient to use a single vehicle; the trip time and the limited capacity of rebalancing trucks should also be taken into account in the optimization model. The extension to a fleet of vehicles requires that stations be grouped into almost independent clusters; then, each vehicle will perform rebalancing only within its associated cluster. This would also allow us to consider the time it takes to perform rebalancing, resulting in a more accurate modeling of the rebalancing costs. Finally, it would be interesting to model the user satisfaction and use more complex behavior patterns, e.g., users taking a bike from a nearby station if the one they choose is empty.
